# Efficacy and Safety of Lumateperone compared to Quetiapine in Indian patients with Bipolar II depression: A subgroup analysis based on prior hypomanic episodes

**DOI:** 10.1192/j.eurpsy.2025.440

**Published:** 2025-08-26

**Authors:** A. Dharmadhikari, P. K. Chaurasia, Y. Patel, D. Choudhary, P. L. Dasud, M. Bhirud, P. S. Meena, F. Shah, G. Ganesan, B. P. S. Rathour, K. Mistry, M. Dutta, A. Ramaraju, S. B. Mangalwedhe, S. G. Goyal, G. Kulkarni, A. Mukhopadhyay, P. Chaudhary, G. T. Harsha, M. Parikh, S. Dey, S. Sarkhel, N. U. Jyothi, A. Kumar, N. K. Sooch, A. Shetty, S. Saha, P. H. Devkare, A. Shetty, D. Patil, P. Ghadge, A. Mane, S. Mehta

**Affiliations:** 1 Shree Ashirwad Hospital, Dombivali; 2 Gangoshri Hospital, Varanasi; 3 VS General Hospital, Ahmedabad; 4 GSVM Medical College, Kanpur; 5 Global 5 Hospital, Navi Mumbai; 6 Dhadiwal Hospital, Nashik; 7 Jawahar Lal Nehru Medical College, Ajmer; 8 Health 1 Super Speciality Hospital, Ahmedabad; 9 Medstar Speciality Hospital, Bangalore; 10 Atmaram Child Care and Critical Care Hospital, Kanpur; 11 Prajna Health Care, Ahmedabad; 12 Om Hospital, Raipur; 13 Harshamitra Super Speciality Cancer Center and research institute, Trichy; 14 Karnataka Institute of Medical Sciences, Hubli; 15 S. P. Medical College & A.G. Of Hospitals, Bikaner; 16 Manodnya Nursing Home, Sangli; 17 Nil Ratan Sircar Medical College and Hospital, Kolkata; 18 GMERS Medical College, Ahmedabad; 19 Rajlaxmi Hospital, Bangalore; 20 B.J. Medical College and Civil Hospital, Ahmedabad; 21 Sparsh Hospital, Bhubaneswar; 22 IPGME&R and SSKM Hospital, Kolkata; 23 Government General Hospital, Guntur; 24 S N Medical College, Agra; 25 Dayanand Medical College & Hospital, Ludhiana; 26 Sun Pharma, Mumbai, India

## Abstract

**Introduction:**

Lumateperone, an atypical antipsychotic drug, has a dual mechanism of action by combination of activity at central serotonin (5-HT2A) and dopamine (D2) receptors.

**Objectives:**

This subgroup analysis of an Indian Phase 3 study was conducted to evaluate the efficacy and safety of Lumateperone 42mg compared to Quetiapine 300mg in treatment of Bipolar II depression when stratified based on prior hypomanic episodes.

**Methods:**

The phase-III, randomized, multi-centric, assessor-blind, parallel-group, active-controlled, comparative, non-inferiority study included Indian patients with Bipolar II depression with moderate severity having a Montgomery-Asberg depression rating scale (MADRS) score ≥20 and Clinical global impression–bipolar version–severity (CGI-BP-S) score ≥4. The study was conducted after receiving regulatory and ethics committee approvals. The patients were randomized (1:1) to either receive Lumateperone 42mg [Test] or Quetiapine 300mg [Comparator] for 6 weeks. The patients were stratified based on number of prior hypomanic episodes in lifetime: Subgroup 1 [S1]: 1 episode and Subgroup 2 [S2]: >1 episode. For efficacy outcomes MADRS score, CGI-BP-S (total score, depression subscore and overall bipolar illness subscore), and Quality of life enjoyment and satisfaction-short form questionnaire (Q-LES-Q-SF) score were evaluated and for safety outcomes treatment emergent adverse events (TEAEs) were assessed. [Clinical trial registration: CTRI/2023/10/058583]

**Results:**

This subgroup analysis included 462 patients, out of which 251 in S1[Test=129; Comparator=122] and 211 in S2[Test=102; Comparator=109]. The baseline demographic characteristics were comparable in between treatment arms across subgroups. The primary endpoint of reduction in MADRS score from baseline to Day 42 in Test arm was non-inferior to Comparator arm in both subgroups [Figure 1] as the upper 95% CI was below the pre-defined margin of 3.0. The reduction of CGI-BP-S (total score, depression subscore and overall bipolar illness subscore) from Day 14 to Day 42 were comparable in both Test and Comparator arms in both subgroups. The improvement in Q-LES-Q-SF score from baseline to Day 42 were comparable in both Test and Comparator arms in both subgroups. The incidence of TEAEs were similar in both treatment arms [S1: Test=37.2% and Comparator=35.2%; S2: Test=31.4% and Comparator=35.8%] and no serious adverse events were reported.

**Image 1:**

**Figure:**
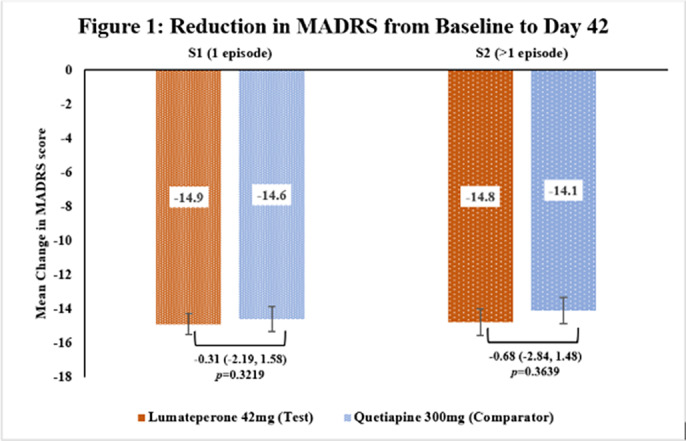

**Conclusions:**

This subgroup analysis demonstrated that Lumateperone 42mg is non-inferior to Quetiapine 300mg in treatment of Bipolar II depression as assessed via MADRS score from baseline to Day 42, irrespective of number of previous hypomanic episodes and both treatments were found to be well tolerated.

**Disclosure of Interest:**

A. Dharmadhikari: None Declared, P. Chaurasia: None Declared, Y. Patel: None Declared, D. Choudhary: None Declared, P. Dasud: None Declared, M. Bhirud: None Declared, P. Meena: None Declared, F. Shah: None Declared, G. Ganesan: None Declared, B. P. Rathour: None Declared, K. Mistry: None Declared, M. Dutta: None Declared, A. Ramaraju: None Declared, S. Mangalwedhe: None Declared, S. G. Goyal: None Declared, G. Kulkarni: None Declared, A. Mukhopadhyay: None Declared, P. Chaudhary: None Declared, G. T. Harsha: None Declared, M. Parikh: None Declared, S. Dey: None Declared, S. Sarkhel: None Declared, N. Jyothi: None Declared, A. Kumar: None Declared, N. Sooch: None Declared, A. Shetty Employee of: Sun Pharma, S. Saha Employee of: Sun Pharma, P. Devkare Employee of: Sun Pharma, A. Shetty Employee of: Sun Pharma, D. Patil Employee of: Sun Pharma, P. Ghadge Employee of: Sun Pharma, A. Mane Employee of: Sun Pharma, S. Mehta Employee of: Sun Pharma.

